# Advances in statistical methods for cancer surveillance research: an age-period-cohort perspective

**DOI:** 10.3389/fonc.2023.1332429

**Published:** 2024-02-09

**Authors:** Philip S. Rosenberg, Adalberto Miranda-Filho

**Affiliations:** Division of Cancer Epidemiology and Genetics, Biostatistics Branch, National Cancer Institute, Bethesda, MD, United States

**Keywords:** cancer surveillance research, Lexis diagram, statistical methods, age-period-cohort model, SEER program

## Abstract

**Background:**

Analysis of Lexis diagrams (population-based cancer incidence and mortality rates indexed by age group and calendar period) requires specialized statistical methods. However, existing methods have limitations that can now be overcome using new approaches.

**Methods:**

We assembled a “toolbox” of novel methods to identify trends and patterns by age group, calendar period, and birth cohort. We evaluated operating characteristics across 152 cancer incidence Lexis diagrams compiled from United States (US) Surveillance, Epidemiology and End Results Program data for 21 leading cancers in men and women in four race and ethnicity groups (the “cancer incidence panel”).

**Results:**

Nonparametric singular values adaptive kernel filtration (SIFT) decreased the estimated root mean squared error by 90% across the cancer incidence panel. A novel method for semi-parametric age-period-cohort analysis (SAGE) provided optimally smoothed estimates of age-period-cohort (APC) estimable functions and stabilized estimates of lack-of-fit (LOF). SAGE identified statistically significant birth cohort effects across the entire cancer panel; LOF had little impact. As illustrated for colon cancer, newly developed methods for comparative age-period-cohort analysis can elucidate cancer heterogeneity that would otherwise be difficult or impossible to discern using standard methods.

**Conclusions:**

Cancer surveillance researchers can now identify fine-scale temporal signals with unprecedented accuracy and elucidate cancer heterogeneity with unprecedented specificity. Birth cohort effects are ubiquitous modulators of cancer incidence in the US. The novel methods described here can advance cancer surveillance research.

## 1 Introduction

Cancer Surveillance Research (CSR) (1) is an observational science of cancer occurrences ascertained in population-based cohorts, notably, cancer registries. CSR is dedicated to tracking cancer incidence and mortality; quantifying cancer differences; characterizing cancer’s natural history and its evolution over time; uncovering etiologic clues; gauging effectiveness of screening and therapy; and informing cancer control programs.

To date, most CSR studies have relied on specialized nonparametric statistical tools that are effective and popular (2, 3). The parametric age-period-cohort (APC) model provides a complementary approach (4–7). Even so, large scale studies covering many populations or outcomes (8, 9) are labor intensive and demand technical expertise, thereby pushing the boundaries of feasibility.

Advances in biostatistics and data science have the potential to usher a ‘golden age’ where high-quality data are universally accessible, and contemporary methods from biostatistics and data science are rapidly and freely deployable. To contribute to this vision, we survey a “toolbox” of newly developed biostatistical methods for analyzing population-based cancer incidence and mortality data. The unique focus of this toolbox is its age-period-cohort perspective.

This is an opportune time to propose such an upgrade. In the United States (US), the cancer landscape has evolved over the last half-century as the US population grew, aged, and changed (10). Throughout this period, the Surveillance, Epidemiology, and End Results (SEER) Program accumulated authoritative population-based data on cancer outcomes (11). Globally, cancer is rapidly rising in many countries (9). Fortunately, the number of high-quality population-based cancer registries has also increased over time (12, 13).

In Section 2, we assemble a panel of examples and illustrate limitations and pitfalls of traditional methods. In Section 3, we present promising new methods that complement the traditional approaches. In Section 4, we provide a summary and outline avenues for future research.

**Figure 1:**
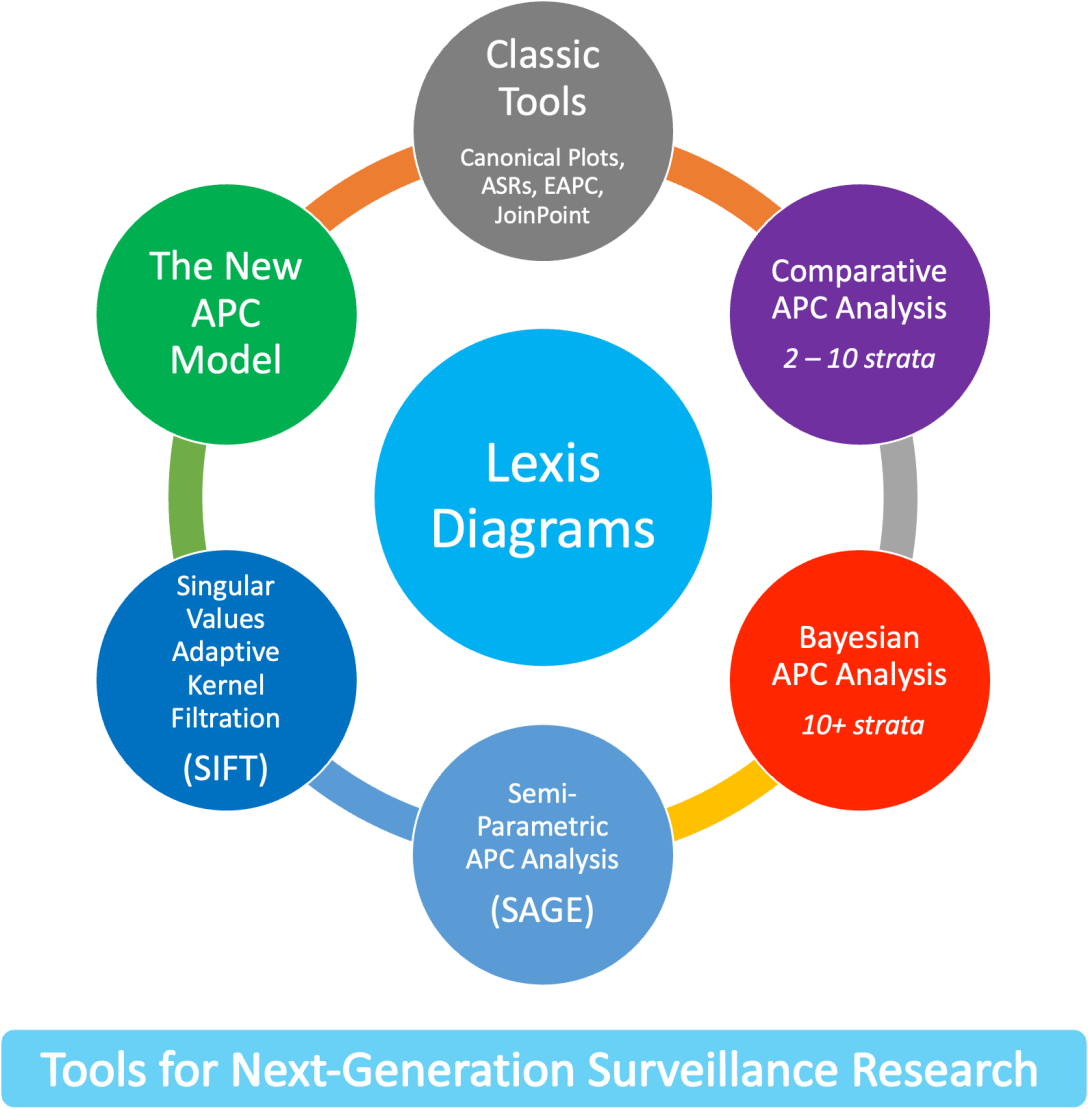
New Tools for Next-Generation Surveillance Research.

The new methods leverage four core principles. First, the Lexis diagram (14) is a fundamental construct that provides a unifying schema for the data. Second, nonparametric smoothing techniques for the Lexis diagram (15, 16) enhance our ability to quantify trends. Third, no analysis of a Lexis diagram is complete without considering the effects of birth cohort: This is most easily accomplished using APC models (6, 17, 18). Fourth, newly developed methods for comparative analysis (19–25) can elucidate heterogeneity between Lexis diagrams ascertained within strata defined by factors such as sex, race and ethnicity, geographic region, and tumor characteristics. We present an overview of these approaches in **Figure 1**.

## 2 Materials and methods

### 2.1 Lexis diagrams

The Lexis diagram (18) is a rectangular grid with binned age groups along one axis and binned calendar periods along the other. Individuals from a surveilled population contribute person-years (number of people and the amount of time at risk) and events (incident cancers, or deaths by cause) to each cell. The observed event counts are modeled as independent Poisson random variables with or without overdispersion. Cells along the diagonals represent persons born in the same period (birth cohorts). Lexis diagrams can be obtained from hundreds of population-based cancer registries worldwide, from the Surveillance, Epidemiology, and End Results (SEER) Program (26), the North American Association of Central Cancer Registries (NAACCR (13)), and the International Agency for Research on Cancer (Cancer Incidence in Five Continents, CI5 (27)).

Using SEER’s Thirteen Registries Database (28), we constructed a panel of 152 cancer incidence Lexis diagrams covering 50 single-years of age (ages 35 – 84), 27 calendar years (1992 – 2018), and 76 single-year birth cohorts (1908 – 1983) for 21 leading cancers in women and men within four race and ethnicity categories: non-Hispanic White (NHW), non-Hispanic Black (NHB), Hispanic (HIS), and Asian and Pacific Islander (API). The 21 cancer sites are: esophagus, stomach, gallbladder, liver, pancreas, colon, rectum, kidney, bladder, leukemia, non-Hodgkin Lymphoma (NHL), myeloma, brain, thyroid, lung, melanoma, breast, ovary, corpus, cervix, and prostate.

### 2.2 Classic methods

**Figure 2:**
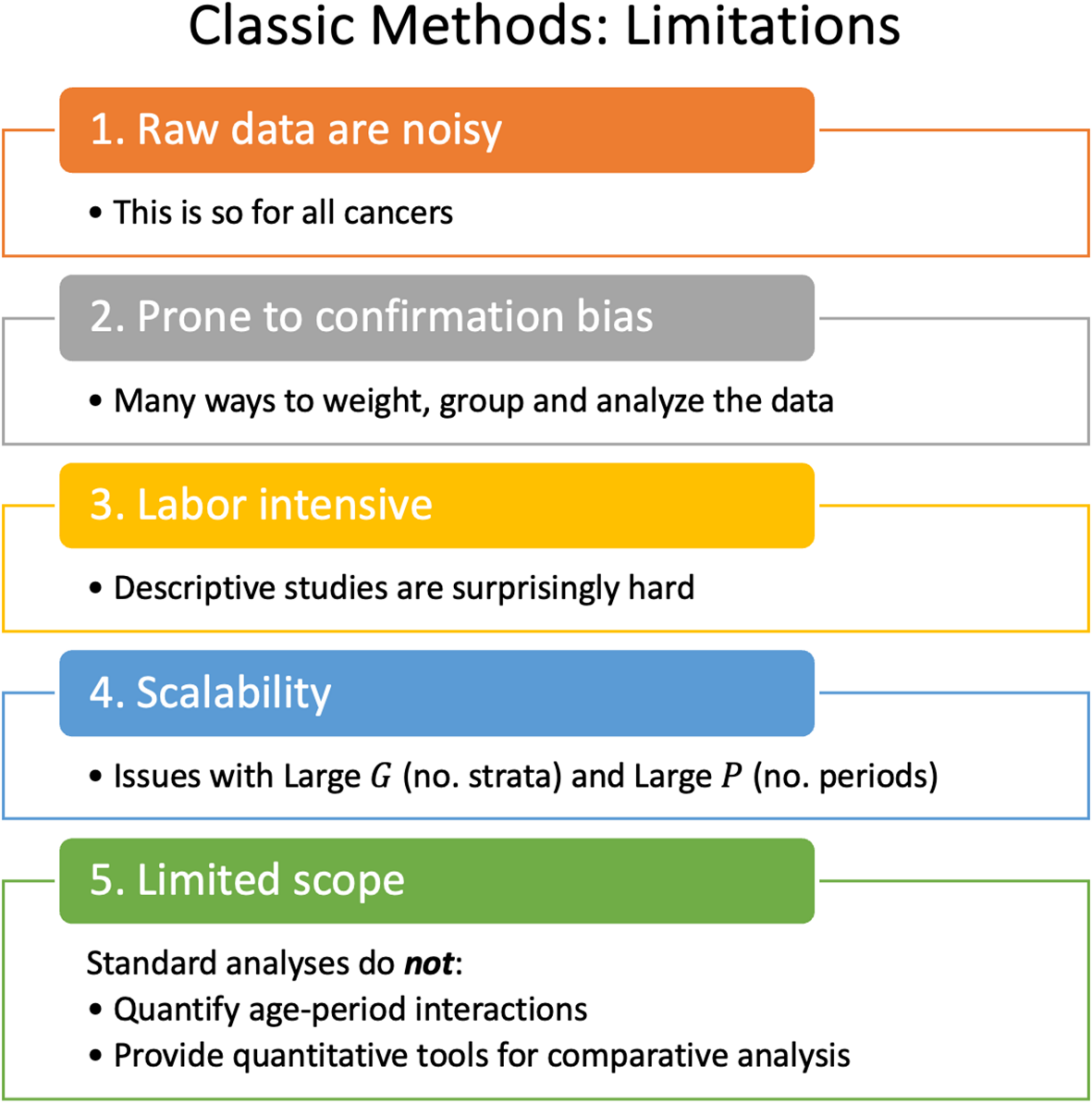
Classic Methods: Limitations.

Lexis diagrams are analyzed using four classic methods: canonical plots for visualization of age-specific rates (29, 30); age-standardized rates (31) (ASRs) for dimension reduction; estimated annual percentage change (EAPC) of the ASRs for trend estimation (32); and JoinPoint analysis for gradient estimation (33, 34), e.g., to identify changes in the EAPC of the ASR over time. These popular statistical tools have limits that warrant attention, summarized in **Figure 2**.

#### 2.2.1 To lump or to split?

The most granular possible Lexis diagrams obtainable from population-based cancer registries encapsulate the rates for single-years of age within single calendar years (1x1s). If the data are sparse, we can bin the 1x1s to 2x2s or 5x5s. The CI5 database (12) provides five-by-ones (5x1s): five-year age groups within single calendar years. The novel methods described in this report require *equal* bin widths for age and period. So, for 5x1s, we must bin the single calendar years into five-year periods or interpolate to single years of age from the age quinquennium within each calendar year. While feasible, interpolation can introduce bias, complicating the interpretation of the results.

Hence, we face a choice: We can analyze 5x5s, 2x2s, or 1x1s. Going one way or the other makes an implicit bias-variance trade-off. Opting to lump may introduce bias, but the granular data are noisy.

#### 2.2.2 ASRs and EAPCs: more than one

There are four widely recognized standard populations (e.g., US 2000 Census, Canadian, WHO World 2000, and Uniform), and four well-posed estimators of trend (32). Essentially all studies select only one of these 16 possibilities. Are conclusions sensitive to this choice?

**Figure 3:**
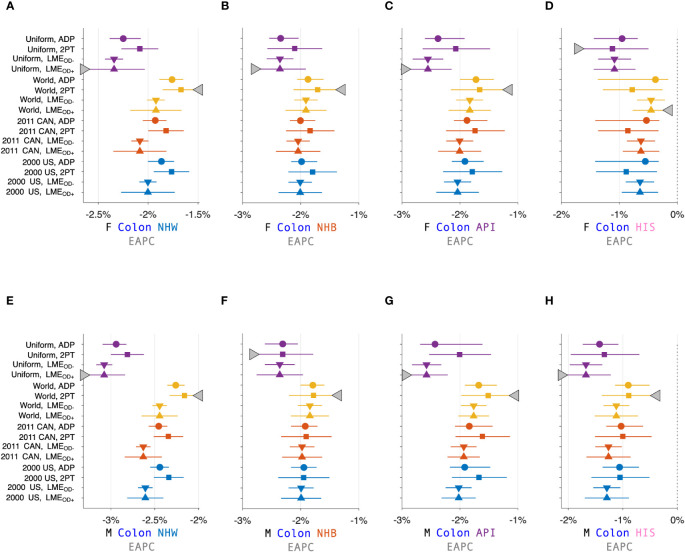
Estimated Annual Percentage Change (EAPC) for Colon Cancer. EAPC for colon cancer stratified by sex and race and ethnicity in Panels **(A–H)**. Each panel presents a Forest Plot of 16 estimators based on four standard populations (Uniform, World, 2011 CAN, and 2000 US) and four trend estimators: Adaptive (ADP); Two-Point (2PT); Linear Model Estimator (LME) without overdispersion ( $LME_{OD-}$ ); and LME with overdispersion ( $LME_{OD-}$ ). Females (F): **(A–D)**. Males (M): **(E–H)**. Non-Hispanic White (NHW) **(A, E)**, non-Hispanic Black (NHB) **(B, F)**, Asian and Pacific Islander (API) **(C, G)**, and Hispanic **(D, H)**. Lowest lower limits and highest upper limits are highlighted by left- and right-facing triangles, respectively.

**Figure 4:**
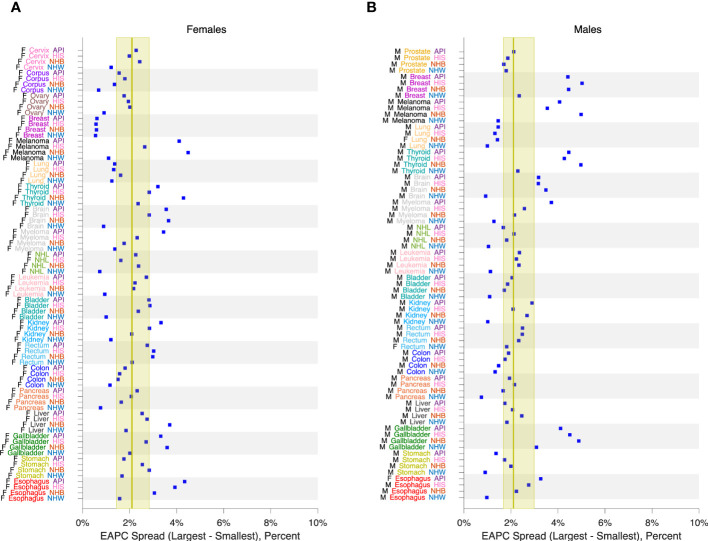
EAPC Spread in 152 Cancers. EAPC spread: Range between lowest lower limit and highest upper limit over 16 EAPC estimators as described in the legend to **Figure 3**. Panel **(A)**, Females. Panel **(B)**, Males. Values by race and ethnicity (API, HIS, NHB, NHW) within cancer sites as labelled. Yellow reference intervals show median and inter-quartile range for 80 Lexis diagrams in females **(A)** and 72 in males **(B)**. Cancer sites: Cervix, Corpus, Ovary, Breast, Melanoma, Lung, Thyroid, Brain, Myeloma, NHL, Leukemia, Bladder, Kidney, Rectum, Colon, Pancreas, Liver, Gallbladder, Stomach, Esophagus, Prostate.

**Figure 3** calculates 16 estimators of EAPC for colon cancer incidence in NHW, NHB, API and HIS women and men. The estimates in each stratum are heterogeneous (Panels A – H), and the EAPC spread – the range between the left- and right-facing triangles – ranges from 1.5 to 2 percent across the panels. Similar heterogeneity is seen across the Cancer Incidence Panel (**Figure 4**): the EAPC spread varies by around 2% on average in both females (Panel A) and males (Panel B). This amount of heterogeneity is substantial, given that EAPCs and EAPC differences in excess of ± 0.5% are generally considered notable. One appeal of the APC Net Drift parameter described in Section 2.3.1 is there is only one.

#### 2.2.3 The problem with JoinPoint is scalability

JoinPoint is a signature method of CSR (33). Whereas the EAPC estimates the *average* rate of change over time, JoinPoint estimates the *gradient*, i.e., the *instantaneous* rate of change. Typically, JoinPoint is applied to age-standardized or age-group-specific (a.k.a. truncated) rates over time (35). JoinPoint can also be used in conjunction with APC models, for example, to identify changes in birth cohort effects. In principle, JoinPoint can be applied to *any* series of *n* observations $y_{i}$ at time point $t_{i},\;i=1,\ldots ,\;n$ , with a full-rank variance-covariance matrix Σ . In the context of CSR, the time series are *equally spaced*.

JoinPoint fits a piecewise linear spline to the data, where the number and locations of the knots, or join-points are estimated from the data. The corresponding gradient curve, a step function, obtains from the *slopes* of the fitted linear spline. To fit a JoinPoint model, we must specify 4 constraints: 1) the minimum number of segments $k_{min}$ , the maximum number of segments $k_{max}$ , the minimum number of time points per segment a , and the maximum number of time points per segment b .

For knot locations restricted to $t_{i},\;i=1,\ldots ,\;n$ , the set of possible JoinPoint models corresponds to the set of doubly restricted integer combinations of n , $RIC\left(n,k_{min},k_{max},a,b\right)$ (36). Efficient formulas and code exist for enumerating RICs (37). As n increases, it becomes increasingly difficult to fit the model without imposing strong restrictions on $k_{max}$ and a , because the RIC numbers become too big.

Suppose we wish to fit a JoinPoint model for 76 single-year birth cohorts, e.g., 1908 – 1983, as in Section 2.1. To fit up to 5 segments each with 10 or more cohorts, JoinPoint must evaluate RIC(76,1,5,10,76)=37,730  models. To allow for up to 10 segments each with 5 or more cohorts – an interesting and plausible scenario – the number is 177,817,540, which is not feasible.

JoinPoint was designed to analyze ASRs for epochs up to several decades long. For this purpose, JoinPoint provides a popular and enduring standard that has recently been improved (34). For applications to longer time series, for example, daily COVID counts, the scalability issue abrogates its appeal as a flexible and adaptive nonparametric estimator of gradients. Fortunately, recent work in this area using stochastic optimization is promising (38).

#### 2.2.4 Standard methods are not designed to detect interactions

Tailored statistical approaches to identify age-period interactions are limited. One exception is the method of Kim et al. (39) for comparing two JoinPoint models.

Savvy epidemiologists have discovered several notable age-period interactions using classic methods alone (40–43). New methods could accelerate the pace of discovery.

### 2.3 The age-period-cohort model

**Figure 5:**
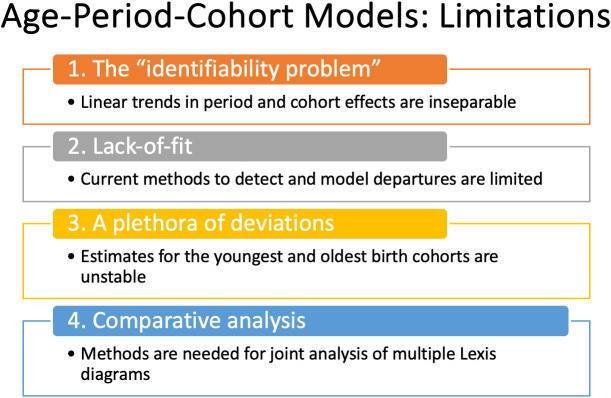
Age-Period-Cohort Models: Limitations.

The APC model is a standard in the field. Fundamentally, it expands the scope of inference. Using the APC model, we can quantify age-period interactions and characterize the longitudinal experience of birth cohorts. Even so, its use in cancer incidence studies has been relatively limited compared to studies that use classic descriptive methods alone, despite freely available software (6, 18). Why is this so? There are several concerns, summarized in **Figure 5**.

Perhaps the biggest are:

1. What about the “identifiability problem”?
2. When is the model appropriate?
3. How can you determine whether the model’s fit is adequate?

#### 2.3.1 Identifiability

The statistical identifiability problem arises because an individual’s year of birth can be determined by subtracting their attained age from the current calendar year. This relationship has an important consequence: when we model event rates in a population, it is impossible to separate the log-linear trend associated with the year of birth, the parameter $\gamma_{L}$ , from the log-linear trend associated with calendar year, the parameter $\pi_{L}$ . We can, however, estimate their sum, $\left(\pi_{L}+\gamma_{L}\right)$ , which is called the Net Drift. In our view, the impossibility of estimating the constituents $\pi_{L}$ and $\gamma_{L}$ in $\left(\pi_{L}+\gamma_{L}\right)$ reflects an intrinsic limitation of observational epidemiologic cohort studies (44). Similarly, the identifiable cross-sectional age trend is $\left(\alpha_{L}+\pi_{L}\right)$ not $\alpha_{L}$ , and the identifiable longitudinal age trend is $\left(\alpha_{L}-\gamma_{L}\right)$ not $\alpha_{L}$ .

*Estimable Functions* (EFs) are linear combinations of model parameters that are invariant with respect to the particular identifiability constraints imposed on the parameters to fit the model (4, 5, 45).

Despite the identifiability problem, the New APC Model (7) provides an expansive array of informative EF based on the intercept μ , the identifiable trend parameters $\left(\alpha_{L}-\gamma_{L}\right)$ , $\left(\alpha_{L}+\pi_{L}\right)$ and $\left(\pi_{L}+\gamma_{L}\right)$ , the global curvature parameters for age, period and cohort, $\theta_{\alpha }$ , $\theta_{\pi }$ , and $\theta_{\gamma }$ , respectively, and the corresponding higher order deviations ${\overset{ˇ}{\gamma }}_{a_{*}},\;{\overset{ˇ}{\gamma }}_{p_{*}}$ , and ${\overset{ˇ}{\gamma }}_{c_{*}}$ . Local Drifts (model-based estimates of the age-specific trends over time) are especially valuable. Please refer to Sections 3 and 5 of the introductory paper for a summary of the parameters, and Table 1 for a summary of essential EF (7).

#### 2.3.2 When is the model appropriate?

“All models are wrong, some are useful” (46). In our context, lack-of-fit (LOF) implies that some birth cohort effects vary over time and age, for example, one generation has higher risk than another for early onset of a cancer, but lower risk for late onset.

In principle, the APC model is well suited for cancer *incidence* if one accepts “the primacy of birth cohort effects.” This concept asserts that: 1) Most cancers (47) have exogenous risk factors (or endogenous risk factors modulated by environmental exposures) and long latency periods from initiation to promotion and progression (48); 2) Exposures in a population typically wax and wane over time. 3) The interplay between biology and tumor natural history induces risk heterogeneity across generations. From this perspective, the APC model is a natural choice for modeling cancer incidence because estimable birth cohort effects quantify net changes in incidence from one birth cohort to the next.

#### 2.3.3 Current methods to assess lack-of-fit are limited

Current methods to assess LOF include estimating over-dispersion parameters, comparing observed and fitted values, and examining residuals (49). In those cases where the LOF is notable, one remedy is to split the rate matrix into blocks within which the LOF is nominal. See the supplement to Best et al. (49) for details. These methods are labor intensive and may not be sensitive, especially for cancers with relatively few events.

## 3 Results: tools for next-generation surveillance research

Recent advances overviewed in **Figure 1** mitigate the limits and concerns summarized in **Figures 2**, **5**. In brief: The SIFT method (Section 3.1 – 3.2) mitigates the limits noted in **Figure 2**. The New APC Model (Section 3.3) eases concerns about identifiability (**Figure 5.1**); SAGE (Section 3.4) addresses worry about lack-of-fit (**Figure 5.2**) and instability (**Figure 5.3**); and sophisticated methods are now available for comparative analysis (**Figure 5.4**).

### 3.1 Sifting through the data

Cancer rates are intrinsically “noisy” (31), and this random variation can mask important signals. The newly developed SIFT (singular values adaptive kernel filtration) method produces smoothed Lexis diagrams with an optimal bias-variance trade-off (50). SIFT incorporates two key innovations. First, for any candidate kernel function, SIFT discards superfluous “high-frequency” basis vectors from the corresponding smoothing matrix based on the bias-corrected Akaike information criterion. Second, because the optimal kernel for any given rate matrix is unknown, SIFT estimates the optimal kernel by model averaging over a panel of candidate kernels with diverse shapes and bandwidths.

SIFT has excellent performance for 1x1 and 2x2 rate matrices (50). Sifted Lexis diagrams are *much* more accurate on a cell-by-cell basis. How much better is it to analyze sifted data versus raw data? We can answer this question more definitively using the Cancer Incidence Panel described in Section 2.1.

For any given Lexis diagram, denote the expected rate per 100,000 person-years in age group a during calendar period p as $E\left(\lambda_{ap}\right)=10^{5}\times \frac{E\left(y_{ap}\right)}{PY_{ap}}$ , where $y_{ap}$ is the observed number of events and $PY_{ap}$ is the corresponding person-years. For the raw data, the Poisson signal-to-noise ratio is $SNR_{Raw}=\frac{E\left(\lambda_{ap}\right)}{Var{\left(\lambda_{ap}\right)}^{1/2}}=E{\left(y_{ap}\right)}^{1/2}$ . Hence, the noise-to-signal percent or relative error is $NSP_{Raw}=100\times SNR_{Raw}^{-1}\%$ .

From the same data, SIFT produces smoothed rates $\lambda_{SIFT}\left(a,p\right)$ and corresponding variances ${\hat{v}}_{SIFT}^{\lambda }\left(a,p\right)$ . Hence, the median estimated NSP for the sifted data is $NSP_{SIFT}=100\times \underset{\left\{\text{cells}\;\left(a,p\right)\right\}}{\text{median}}\frac{{\left[{\hat{v}}_{SIFT}^{\lambda }\left(a,p\right)\right]}^{1/2}}{\lambda_{SIFT}\left(a,p\right)}\%$ .

**Figure 6:**
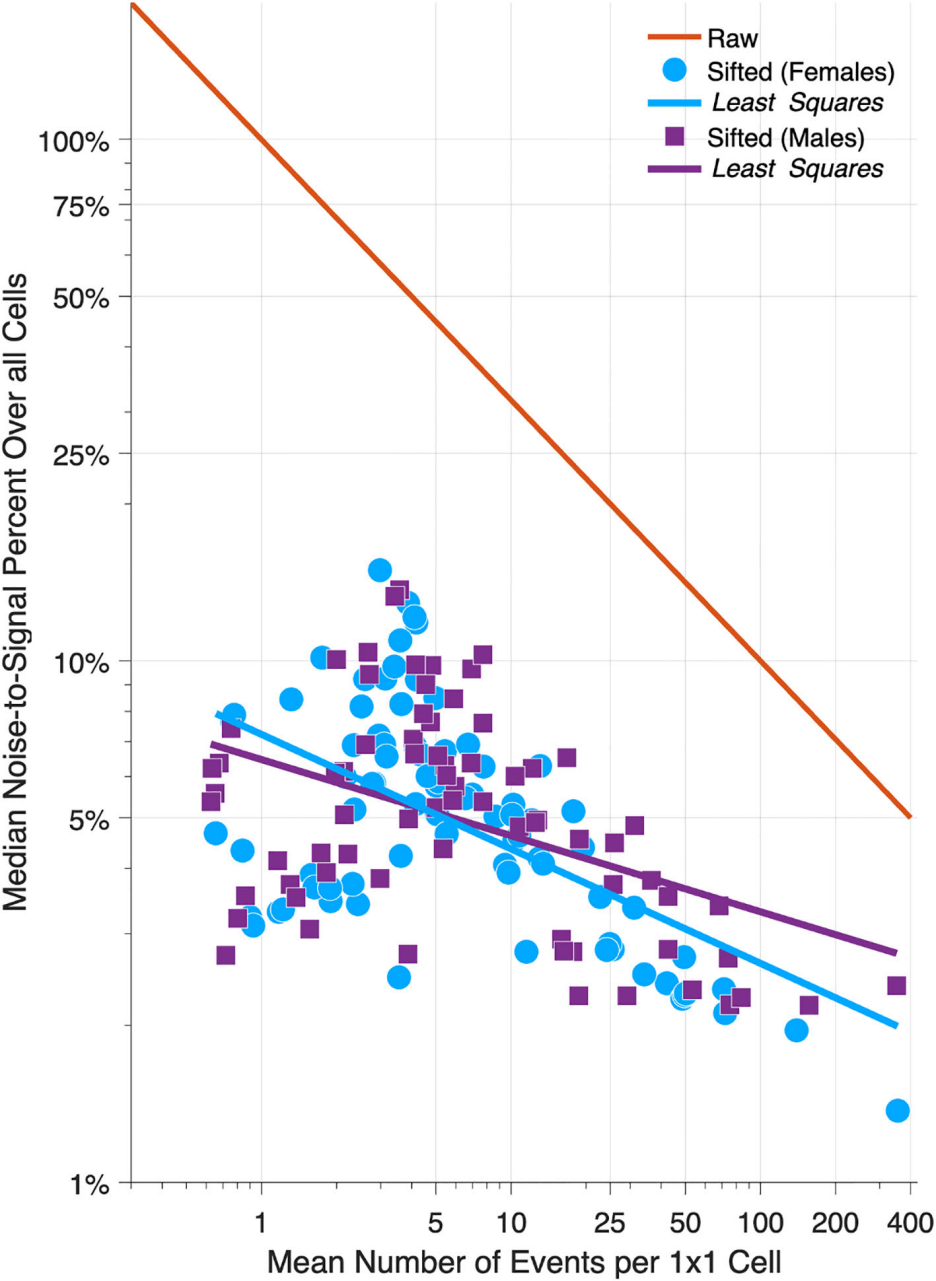
Relative Error in 152 Cancers: Raw versus Sifted Data. Scatter Plot of median noise-to-signal percent versus mean number of events per cell for 80 Lexis diagrams in females (light blue circles) and 72 in males (magenta squares). Light blue and magenta dashed lines: least squares fit. Solid red line: reference curve for raw data assuming Poisson error. Data are plotted on a log-log scale.

**Figure 6** compares NSPs for raw 1x1 data (solid red line) versus sifted data (females, light blue circles; males, magenta squares) for all 152 Lexis diagrams in the Cancer Incidence Panel. NSPs are plotted versus the Lexis diagram’s mean number of events per cell on a log-log scale. For typical 1x1 Lexis diagrams with around 5 events per cell, the NSP is ~50% for the raw data versus ~5% for the sifted data – a 90% reduction. As indicated by the least squares lines for Females (light blue line) and males (magenta line), substantial reductions are expected regardless of the mean number of events per cell. On average, The NSP was reduced by 86% across the panel.

Suppose we eschew sifting, and instead chunk the data from 1x1s to 5x5s. Chunking will indeed reduce the NSP – by 80% - almost as much as SIFT. To see this, compare the red reference line when the mean number of events is 1 versus 25, or 5 versus 125, etc. Unfortunately, we lose temporal resolution. By aggregating 25 cells into one, we throw away four-fifths of our information about age and period effects (one 5-year time point versus five 1-year time points), and eight-ninths of our information about birth cohort effects (1 diagonal in a 1x1 cell versus 9 diagonals in a 5x5 cell). Fortunately, as demonstrated in **Figure 6**, there is no need to do so.

For 5x1 data, rather than chunking up to 5x5s, one might consider interpolating down to 1x1s (51). In our view, this approach merits development: At this point, the optimal interpolation scheme remains unclear.

### 3.2 An abundance of features

A *Feature* is a linear or log-linear combination of the rates. The class of features includes averages, gradients, and trends, in any combination (50). ASRs and EAPCs are features, as are the curves graphed in canonical plots. Features can be calculated from observed data or sifted data. A key point is, Features calculated from sifted data are much more accurate. Furthermore, one way to overcome the scalability issue of the JoinPoint approach (Section 2.2.3) is to extract empirical gradients from the sifted data.

Features can also be calculated from fitted rates obtained via APC models. The essential distinction between Features and EFs is, Features describe expected values of *observed* rates, whereas EFs are estimated from model parameters and therefore describe expected values of *adjusted* rates.

### 3.3 Best practices for APC analysis

Despite the limitations noted in Section 2.3, the APC model greatly expands the scope of inference. When birth cohort effects are present time trends *necessarily* vary by age (7). Since many, perhaps most cancers are influenced by birth cohort effects (Sections 2.3.2 and 3.4), this implies that ASRs and ASR features (EAPC, JoinPoint) at best describe the average trend, which may not provide a reasonable summary of the trends within any given age group. In our view, one should *always* examine either Local Drifts (an EF) or age-specific temporal trends (a Feature; Section 3.2).

When the effects of LOF are modest, one can emphasize conclusions based on EFs, including Local Drifts. Indeed, under the model, Local Drifts are a *consequence* of changes in the gradient of the Fitted Cohort Pattern (FCP; the rate at arbitrary reference age $a_{0}$ in each birth cohort). Hence, the latter provide an *explanation* for the former.

**Figure 7:**
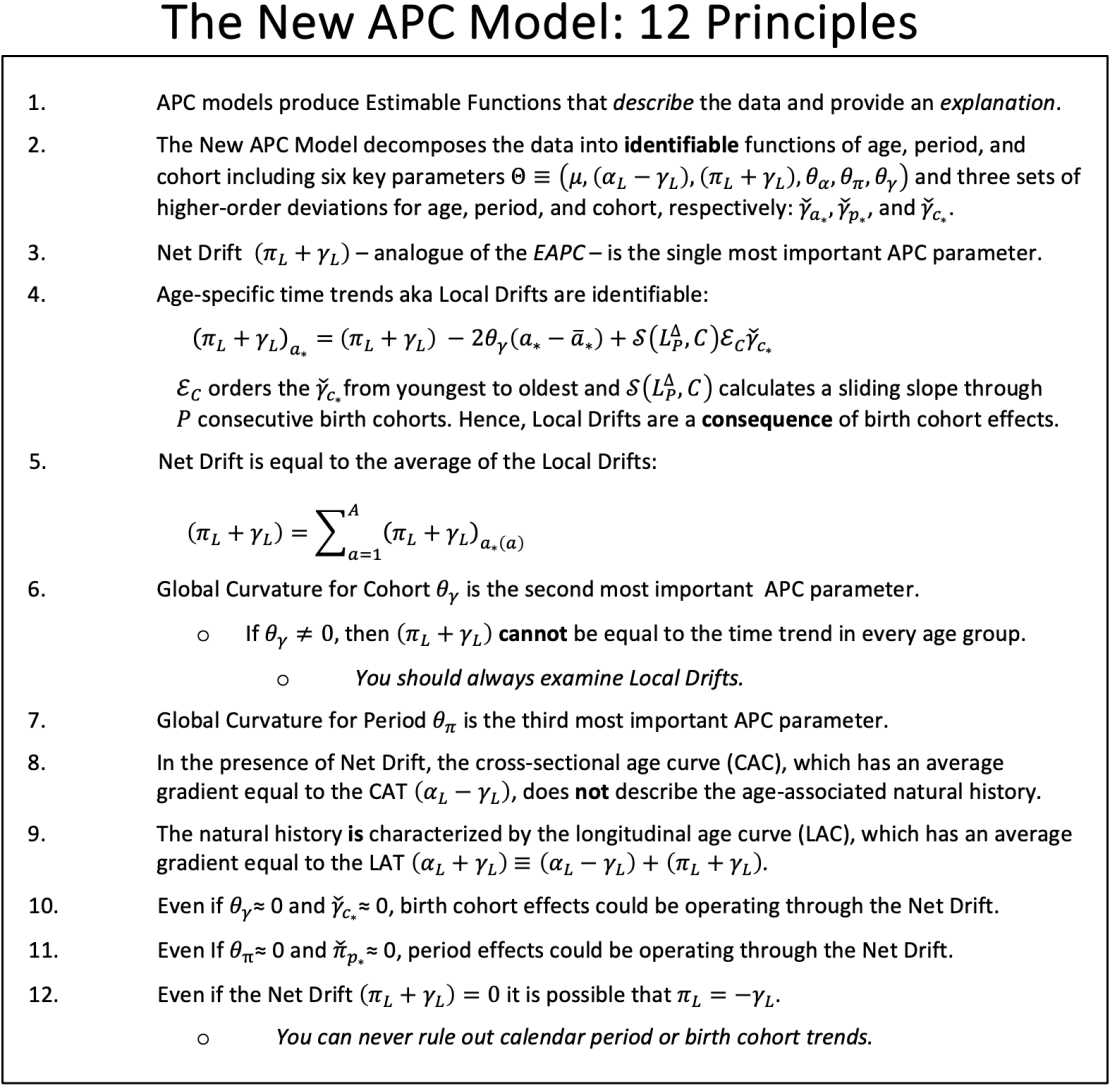
The New Age-Period-Cohort Model: 12 Principles.

In 1987, Clayton and Schifflers (52) presented a popular “checklist” for fitting classic APC models. In **Figure 7** we present a checklist for interpreting model outputs from the New APC Model. A key distinction is our checklist puts Local Drifts front and center.

### 3.4 Semi-parametric age-period-cohort analysis

We have in hand two powerful and complementary approaches – the New APC Model and SIFT – parametric and nonparametric. Can we combine them?

One natural way to do so is to de-noise the raw data using SIFT, and then partition the sifted values into a component arising from the APC model plus a residual component that represents the LOF. We will call this procedure SAGE, an acronym for Semi-Parametric Age-Period-Cohort Analysis. **Algorithm 1** presents the details.

Algorithm 1 **Semi-Parametric Age-Period-Cohort Analysis (SAGE).**

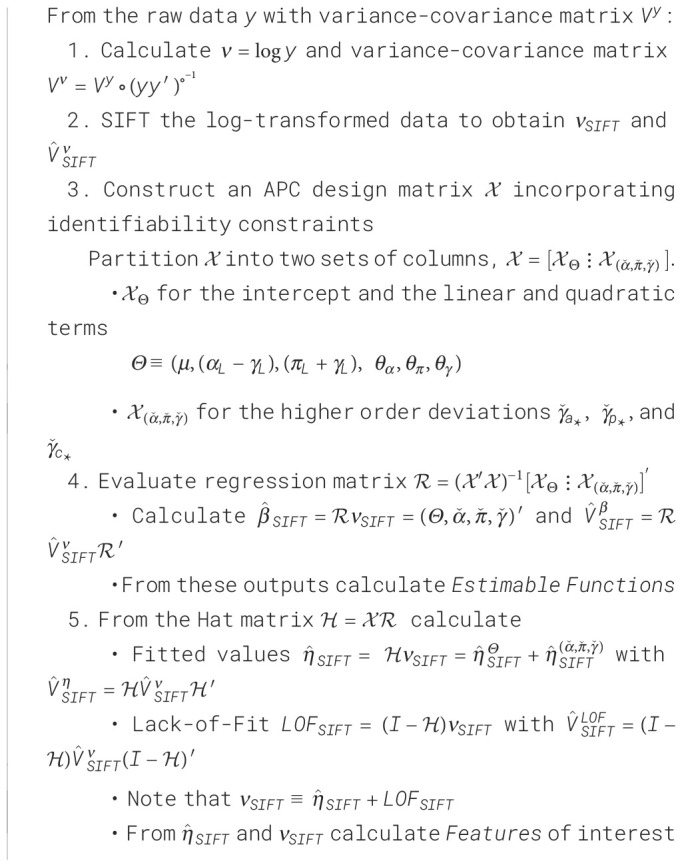

SAGE advances our understanding of the data in two ways. First, we are better able to examine LOF and gauge its impact on Features. Second, when the model appears adequate, we can draw conclusions from the EF. These estimates will be smoother and have narrower confidence limits than corresponding estimates obtained by fitting the APC model to the raw data.

**Figure 8:**
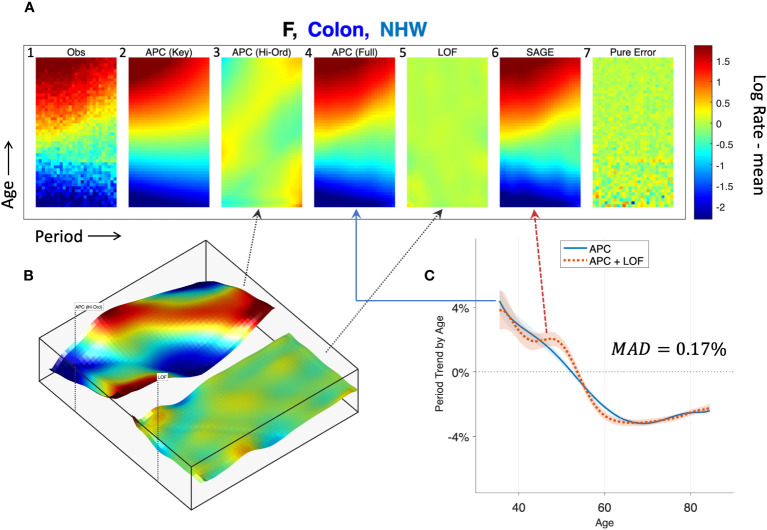
Semi-parametric Age-Period-Cohort (SAGE) Analysis. SAGE analysis of colon cancer incidence in Non-Hispanic White Females. **(A)**: Heat Maps of raw data **(A.1)**, pure error **(A.7)**, and decomposition of sifted data **(A.6)** into APC model components **(A.2–A.4)** and Lack-of-Fit (LOF; **A.5**). **(B)**: Surface Plots of APC higher-order deviations (left panel) versus LOF (right panel). **(C)**: Age-specific period trends with (dashed red curve) and without (solid blue curve) LOF. The median absolute deviation (*MAD*) between the curves in 0.17%.

To illustrate, we applied SAGE to colon cancer incidence among NHW women and visualized the outputs using heat maps (**Figure 8**, Panel A). Panel A.1 shows the raw data, A.6 the sifted values (“SAGE”), and A.7 the SIFT residuals (“Pure Error”). Panels A.2 – A5 present the partitions described in Step 5 of the SAGE algorithm. The full APC model (A.4) is the sum of contributions from the key parameters (A.2) and higher-order deviations (A.3).

Panel B plots higher-order deviations and LOF using surface plots to better gauge their relative magnitudes. The former is substantially larger. Panel C shows the estimated period trend by age from the APC model (solid blue) and the APC model plus LOF (dash red). There are small gaps between the curves, especially at around age 50, when the model appears to under-estimate the empirical trend by around 0.5% per year. The median absolute deviation (MAD) between the parametric and nonparametric curves is 0.17% per year.

**Figure 9:**
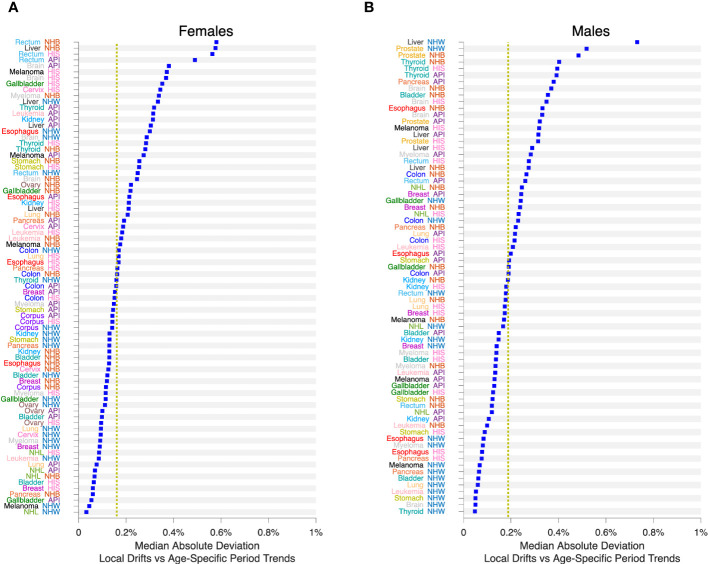
Impact of Lack-of-Fit (LOF) on Local Drifts in 152 Cancers. Median Absolute Deviation ( MAD ) between Local Drifts and Age-Specific Period Trends from SAGE analysis. See the Legend to **Figure 8** for details. **(A)**, Females. **(B)**, Males. Values by race and ethnicity (API, HIS, NHB, NHW) within cancer sites as labelled. Yellow reference lines show median of MAD values for Females and Males.

We applied SAGE to all Lexis diagrams in the Cancer Incidence Panel. Comparing the period trends by age (APC model versus APC Model plus LOF), the MAD never exceeded 0.6% per year in Females (**Figure 9A**) or 0.8% per year in Males (**Figure 9B**). On average, the MAD was 0.16% per year in Females and 0.19% per year in Males.

**Figure 10:**
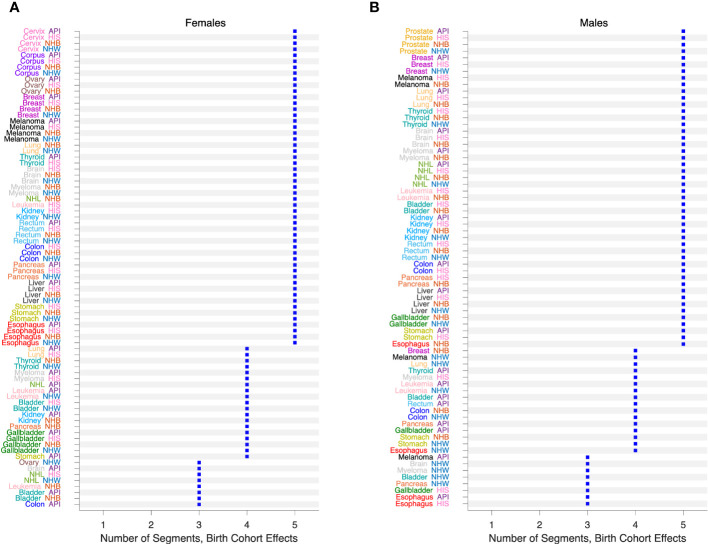
Birth Cohort Effects in 152 Cancers. Number of segments identified by JoinPoint analysis of Fitted Cohort Patterns from SAGE. **(A)**, Females. **(B)**, Males. Values by race and ethnicity (API, HIS, NHB, NHW) within cancer sites as labelled.

We also fitted JoinPoint models to the FCPs from SAGE, allowing up to 5 segments each with 10 or more birth cohorts. **Figure 10** presents the number of segments identified by the JoinPoint permutation test (33) in Females (**Figure 10A**) and Males (**Figure 10B**). In every case, the number of segments was 3 or more.

### 3.5 Comparative age-period-cohort analysis

The APC model describes a single Lexis diagram. Most studies involve ensembles of G≥2 Lexis diagrams defined by strata such as sex, race and ethnicity, geographic region, tumor characteristics, etc. A key goal is to compare and contrast EF *between* strata. One can think of the strata as covariates. However, the Lexis diagram can be analyzed on four different time scales (6), and the event rates can be proportional with respect to one time scale but not the others (53).

Recently, we developed a comparative method that can identify whether the stratum-specific hazard rates in an ensemble of G≥2 Lexis diagrams are *proportional* overall, or within calendar periods, age groups, or birth cohorts (25). Proportionality imposes meaningful constraints on the stratum-specific EF. For example, when the hazard rates are proportional within calendar periods, the Local Drifts for each stratum are all equal. Alternatively, when the hazards are proportional within age groups, the stratum-specific Local Drifts are parallel. Such constraints can highlight important signals that otherwise might be missed by inspection of outputs from separate models.

**Figure 11:**
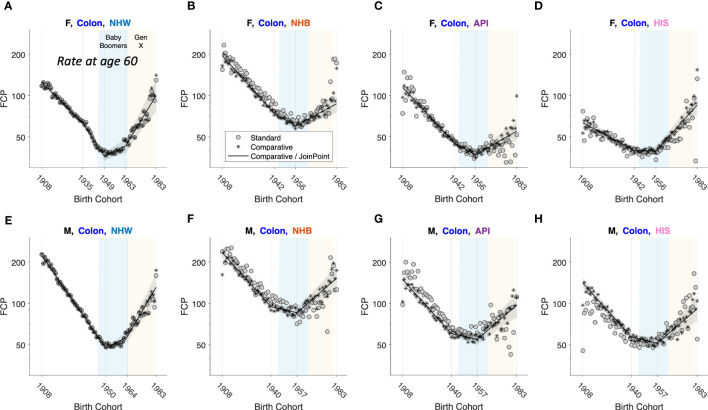
Fitted Cohort Patterns (FCPs) for Colon Cancer. FCP: Expected rate at age 60 by birth cohort. Separate APC models (grey circles); joint APC model identified by exploratory comparative analysis (stars); JoinPoint analysis of the joint model (black curves with grey pointwise 95 percent confidence limits). Females (F): **(A–D)**. Males (M): **(E–H)**. Non-Hispanic White (NHW) **(A, E)**, non-Hispanic Black (NHB) **(B, F)**, Asian and Pacific Islander (API) **(C, G)** and Hispanic (HIS) **(D, H)**. In each panel, x-axis ticks show estimated join-points. Baby-Boomer (blue) and Gen-X (yellow) birth cohorts are highlighted.

To illustrate, we carried out an exploratory comparative analysis of colon cancer incidence by sex, race, and ethnicity. The analysis partitioned the 8 strata into 4 subsets: non-proportionality in NHW women and men, age proportionality in NHB, API and HIS women, and absolute proportionality in NHB, API and HIS men. From these partitions we extracted FCPs and ran JoinPoint models (**Figure 11**). In each stratum, colon cancer incidence bottomed out among Baby Boomers (1946 – 1964 birth cohorts), then increased year-over-year among members of Generation X (1965 – 1980 birth cohorts).

It is computationally feasible to evaluate partitions when the number of strata is small to moderate, 2≤G≤10 . When G>10 , Bayesian methods provide a valuable approach. Bayesian spatial age-period-cohort analysis is one promising application (21). Bayesian methods can be used to characterize the distribution of Features. In practice it appears essential to take birth cohort effects into account (**Figure 10**). One way to do so is to carry out the Bayesian analyses separately within age strata (22).

### 3.6 Cancer forecasts

Forecasts of cancer incidence obtained from APC models are popular because the underlying assumptions of the model are often reasonable (Section 2.3.2) (54). APC-based forecasts extrapolate parameter estimates from observed to future age and period cells (44, 54). Consequently, Incidence forecasts are EF. Different scenarios can be modeled by varying the extrapolation scheme used to account for future period and cohort deviations. Best et al. (49) extrapolate future period effects using the global curvature for period, and future cohort effects using the most recent segment from a join-point analysis of the FCP, i.e., by extrapolating from the experience of the youngest observed birth cohorts.

## 4 Discussion

The novel statistical approaches we describe here do not replace classic analytical tools and methods for cancer rates: they build upon them. Each method starts with one or more Lexis diagrams. SIFT and SAGE increase accuracy by leveraging the power of contemporary nonparametric smoothing. Indeed, SIFT is our recommended smoother for 1x1 and 2x2 data because it offers remarkable increases in precision across a broad spectrum of cancers (**Figure 6**). The New APC Model and SAGE elucidate birth cohort effects: The latter provides appealingly smooth Estimable Functions (EF). Comparative APC Analysis ascertains cancer heterogeneity across ages, periods, and birth cohorts for a small to moderate number of strata, and Bayesian methods when the number of strata is moderate to large. Taken together, the new methods summarized in **Figure 1** mitigate the limits highlighted in **Figures 2**–**5**.

With these new tools in hand, our ability to detect fine-scale temporal signals in granular data with one- or two-year age and period intervals is greatly enhanced. Indeed, smoothing Lexis diagrams up front using a contemporary non-parametric procedure such as SIFT has compelling advantages. Accuracy is *greatly* increased (**Figure 6**), and you can extract all of the standard Features from the sifted data (e.g., canonical plots, ASRs, EAPC, and JoinPoint). You can also extract novel Features defined by averages, trends, and gradients. One particularly valuable Feature is the sifted estimates of the age-specific trends over time, a model-free analogue of the APC Local Drifts.

The APC Model provides a conceptual framework for interpreting cancer incidence based on a principle we call “the primacy of birth cohort effects”. The APC model can be applied to cancer mortality, with the proviso that trends in mortality reflect changes in both cancer incidence and cancer survival. For mortality analysis it is especially crucial to assess LOF because many treatments are used to a greater or lesser extent according to the patient’s age at diagnosis.

The SAGE method illustrated in **Figure 8** provides a valuable new tool for “stress-testing” an APC model. If the LOF is large relative to the higher-order deviations, one can step away from model-based EF and base conclusions on Features, which are model-free constructs. This strategy improves the overall reliability of the analysis.

Using SAGE, we surveyed 152 cancer incidence Lexis diagrams across 21 leading sites in men and women in four race and ethnicity groups. The LOF had remarkably little impact on the Local Drifts (**Figure 9**). This is not surprising if the underlying expected rates are smooth functions, which is a standard assumption. In that case, it is straightforward to show that the 6 key parameters of the New APC Model describe a second-order Taylor expansion of the Lexis diagram around the middle cell located at $\left({\bar{a}}_{*},{\bar{p}}_{*}\right)$ . Furthermore, given the “primacy of birth cohort effects,” it is not entirely unexpected that birth cohort effects are statistically significant in every case (**Figure 10**).

When birth cohort effects are present – which, for US incidence, appears to be most of the time – the EAPC **cannot** represent the time trend in every age group. Consequently, unless the LOF is substantial, one should always examine birth cohort effects. This is easily done using the New APC Model, but it is much harder to do so using canonical plots or other classic descriptive methods. This is because we observe the oldest cohorts only at older ages and the youngest cohorts only at younger ages: the data are not balanced over cohorts. For the practitioner, our synopsis of “best practices” (**Figure 7**) should provide a handy “cheat sheet” for applications using the New APC Model or SAGE.

Frequentist statistical methods are now available for comparative studies with a small to moderate number of stratum-specific Lexis diagrams, two through around 10, and Bayesian methods when the number of strata is larger (10 up to several hundred). As illustrated in **Figure 11** for colon cancer, Comparative Analysis can identify patterns and signals in birth cohort effects within and between strata that would otherwise be difficult or impossible to detect. Using this approach, we discovered that members of Generation X born between 1965 – 1980 are at increased risk of colon cancer compared to Baby Boomers born between 1946 – 1964. This unfavorable trend was seen across both sexes and in all four race and ethnicity groups.

For each new tool in **Figure 1**, the requisite statistical software is now available or soon will be. It is now technically possible to integrate advances in statistical methodology and data science and put powerful new tools in the hands of cancer surveillance researchers. Doing so could facilitate a ‘golden age’ in CSR. Furthermore, these tools can be combined in novel ways, effectively making new tools. Feature extraction from sifted data (50) and SAGE (**Figure 8**) are two examples.

Whereas forecasts of cancer *incidence* are EF and therefore fall within the purview of the methods summarized in **Figure 1**, forecasts of cancer *burden* – the absolute numbers of new cases – requires a combination approach that integrates population forecasts from the Census Bureau with incidence forecasts from APC models (49, 54–56). In principle, cancer *prevalence* (past, current, and future numbers of persons living with a cancer) can also be estimated using combination methods that integrate survival analysis of cancer cases with APC models of cancer incidence. This would require a separate toolbox of survival methods including cause-specific hazard functions (57, 58) and cumulative incidence of competing risks (59, 60).

Knowing that something is possible does not make it happen – that will require serious work in the area of implementation science, for example, to accelerate computational algorithms for SIFT, SAGE, and Comparative Analysis, scale up the JoinPoint method for longer time series, streamline access to data using Findable, Accessible, Interoperable, Reusable (FAIR) principles (61), and develop interfaces that integrate FAIR data, analysis tools, and workflows (62). In light of recent increases in cancer incidence occurring in the US (63) and globally (9), we believe such efforts are warranted to advance cancer control and cancer research.

## Data availability statement

Publicly available datasets were analyzed in this study. This data can be found here: https://seer.cancer.gov/data/.

## Ethics statement

Our analysis is based entirely on publicly available data.

## Author contributions

PR: Conceptualization, Formal analysis, Methodology, Software, Visualization, Writing – original draft, Writing – review & editing. AM-F: Conceptualization, Data curation, Formal analysis, Methodology, Software, Visualization, Writing – review & editing.
